# Evidence-Based Diagnostic and Therapeutic Methods for Thoracic Outlet Syndrome: A Systematic Review

**DOI:** 10.7759/cureus.108241

**Published:** 2026-05-04

**Authors:** Akshaya V Joshi, Sandeep Shinde, Neha S Chaudhary, Sachin Chaudhary

**Affiliations:** 1 Department of Musculoskeletal Physiotherapy, Krishna College of Physiotherapy, Krishna Vishwa Vidyapeeth (Deemed to be University), Karad, IND; 2 Department of Musculoskeletal Sciences, Krishna College of Physiotherapy, Krishna Vishwa Vidyapeeth (Deemed to be University), Karad, IND; 3 Department of Neuroscience, Datta Meghe College of Physiotherapy, Nagpur, IND; 4 Department of Cardiovascular and Respiratory Physiotherapy, Datta Meghe College of Physiotherapy, Nagpur, IND

**Keywords:** brachial plexus, musculoskeletal disorders, posture, surgical decompression, thoracic outlet syndrome

## Abstract

Thoracic outlet syndrome (TOS) is a condition caused by the compression of neurovascular structures within the thoracic outlet. The primary objective of this systematic review is to evaluate evidence-based diagnostic and therapeutic methods for TOS, with a specific focus on physiotherapy-based interventions. Secondly, this study aims to synthesize recent academic findings to provide medical professionals with a contemporary perspective on effective treatment protocols and to highlight gaps in the existing literature warranting further research. Following the PRISMA framework, a digital search of major platforms, including PubMed, MEDLINE, and Google Scholar, was conducted for literature published up to April 2026. Ten articles met the inclusion criteria, which focused on neurogenic, arterial, and venous subtypes and prioritized physiotherapy-based interventions. Methodological quality was assessed using validated tools such as QUADAS-2, AXIS, and ROBINS-1. The results demonstrate that structured physiotherapy, including postural correction, stretching of the scalenes and pectoralis minor, scapular stabilization, and neural mobilization, is highly effective as a primary conservative treatment for reducing pain and improving upper limb function. Intensive inpatient rehabilitation programs also showed significant, sustained improvements in strength and functional capacity. Additionally, the review highlights that persistent symptoms are often influenced by psychosocial factors such as central sensitization and kinesiophobia, necessitating a multidisciplinary, biopsychosocial approach to management. Despite these findings, the review identifies a significant lack of standardized diagnostic and therapeutic protocols. The study concludes that early identification and the integration of pain neuroscience education into standardized rehabilitation protocols are essential for optimizing patient outcomes and preventing symptom recurrence.

## Introduction and background

Thoracic outlet syndrome (TOS) is a multifactorial disorder resulting from pressure on the neurovascular structures within the thoracic outlet, an anatomical passage extending from the intervertebral foramina to the lower border of the axilla. The thoracic outlet is defined by several anatomical markers: the anterior and middle scalene and the first rib form its medial border, while the clavicle and pectoralis minor provide the anterior limit. Posteriorly, the region is enclosed by the scapula and upper trapezius, extending laterally toward the axilla. Inside this narrow corridor, the brachial plexus is situated between the middle and anterior scalene muscles. The subclavian artery travels behind the anterior scalene, whereas the subclavian vein is positioned in front of it. Such a compact structural layout increases the risk of nerve and vessel constriction in various disease states or under adverse mechanical stress [1].

TOS is broadly classified into neurogenic (nTOS), arterial (aTOS), and venous (vTOS) types. nTOS is the most frequent form of TOS, occurring in more than 90% of patients, whereas aTOS and vTOS account for roughly 1% and 3-5% of the population, respectively [2]. nTOS is typically characterized by upper-limb pain, sensory deficits such as numbness or paresthesia, and functional impairments such as weakness and poor postural alignment. As the condition becomes more severe, clinicians may notice hand muscle wasting and significant intrinsic weakness. Electrophysiological assessments are often utilized in these cases to provide data that supports the clinical diagnosis. When repetitive arm elevation causes the subclavian or axillary artery to become constricted, it leads to the development of aTOS, characterized by symptoms of restricted blood flow. vTOS is characterized by venous congestion, swelling, cyanosis, and, in severe cases, thrombosis of the upper limb veins, presenting with heaviness and visible venous distension [3,4].

The pathophysiology of TOS is multifactorial, involving both structural and functional components. Reduced space within the thoracic outlet is often driven by anatomical variations, such as congenital cervical ribs, as well as muscular issues, such as hypertrophy or excessive tone in the scalene and pectoralis minor groups. Furthermore, constriction of the interscalene and costoclavicular intervals contributes significantly to this narrowing [1]. Functional impairments, such as scapular protraction, rounded shoulders, thoracic kyphosis, and forward head posture, act as significant aggravating factors that increase neurovascular entrapment. Repetitive overhead activities and occupational or sports-related postures may also raise the likelihood of developing TOS. These factors collectively contribute to intermittent or sustained compression, leading to the characteristic clinical manifestations of the syndrome [3].

Because the symptoms of TOS often mimic those of other orthopedic or nerve-related conditions, reaching a definitive diagnosis remains a complex task for clinicians. Effective diagnosis of TOS necessitates the exclusion of similar upper limb pathologies, such as carpal tunnel syndrome and cervical radiculopathy, which share overlapping symptomatic features [1-3]. Clinicians can differentiate the two by observing symptom triggers: cervical radiculopathy is frequently worsened by spinal movement and is confirmed by a positive Spurling's test, whereas TOS manifestations are primarily linked to the position of the upper limb. Similarly, peripheral nerve entrapment syndromes can mimic both distal sensory and motor symptoms seen in nTOS. Therefore, a thorough clinical evaluation, including a detailed history and physical examination, remains the cornerstone of diagnosis, with imaging modalities and electrodiagnostic studies used when necessary [4].

A variety of specialized maneuvers have been developed to aid in the diagnosis of TOS; nonetheless, the reliability of these tests is frequently limited by their inconsistent accuracy. Structural irregularities can be detected using various imaging modalities, such as magnetic resonance imaging, computed tomography, and ultrasound. For cases involving vTOS, Doppler assessments are particularly beneficial for evaluating blood flow [5].

The condition's type and severity determine management of TOS. Conservative treatment remains the first-line approach, particularly for nTOS. Physiotherapy plays a central role and includes postural correction, stretching of tight musculature, such as the scalenes and pectoralis minor, strengthening of scapular stabilizers, neural mobilization techniques, ergonomic modifications, and patient education [1-4]. Pharmacological management, including nonsteroidal anti-inflammatory drugs and muscle relaxants, may be used to alleviate pain and inflammation. In selected cases, injections such as corticosteroids or botulinum toxin may be considered. For vTOS complicated by thrombosis, anticoagulation or thrombolytic therapy is indicated, while aTOS may require more aggressive vascular intervention [6].

When conservative interventions prove ineffective, surgical decompression is indicated for patients presenting with substantial neurological deficits or vascular compromise. Procedures such as first rib resection, scalenectomy, cervical rib excision, or the release of fibrous bands aim to decompress the neurovascular bundle. By alleviating this pressure often through minimally invasive techniques, surgery effectively mitigates pain and paresthesia, resolves vascular complications, and enhances the patient's overall quality of life in severe or progressive cases [1-4]. Following any surgical intervention, targeted postoperative rehabilitation remains crucial for restoring limb function and preventing symptom recurrence [3].

Beyond structural interventions, recent literature underscores the necessity of a multidisciplinary, biopsychosocial framework for TOS management. Psychological factors, including kinesiophobia, pain catastrophizing, and central sensitization, heavily influence clinical outcomes and pain perception. Because the persistent discomfort of TOS stems from both localized musculoskeletal dysfunction and central nervous system alterations, effective recovery protocols must integrate structured, stepwise physical rehabilitation with comprehensive pain neuroscience education [1-5].

Despite advances in understanding and management, the diagnostic evaluation and treatment of TOS remain inconsistent due to variability in clinical practice and the absence of standardized protocols. Studies have highlighted differences in physiotherapy approaches, including variations in exercise selection, dosage, and progression. This inconsistency underscores the need for evidence-based guidelines to standardize care and improve patient outcomes.

Given these challenges, the present systematic review aims to evaluate evidence-based diagnostic and therapeutic methods for TOS, with a focus on physiotherapy-based interventions. Drawing on recent academic findings, this analysis aims to offer medical professionals a contemporary perspective on effective treatment protocols while highlighting gaps in the existing body of work that warrant further study.

## Review

Methodology

Study Design

To maintain high levels of clarity and scientific integrity, this systematic review followed the Preferred Reporting Items for Systematic Reviews and Meta-Analyses (PRISMA) framework during the data collection and reporting phases [7]. The systematic review was registered with PROSPERO (Registration No. CRD420261380206; registered on 27 April 2026).

Search Strategy

This systematic analysis evaluates current protocols for identifying and managing TOS, prioritizing studies that provide a strong evidentiary basis for clinical practice. Research was gathered through a digital search across several major platforms, including Google Scholar, ResearchGate, PEDro, MEDLINE, and PubMed, covering all relevant articles published through April 2026. MeSH terms and free-text keywords such as “Thoracic Outlet Syndrome,” “Brachial Plexus,” “Compression,” “Special Tests,” “Posture,” “Stretching,” and “Upper Limb Tension Test” were employed. In addition to the electronic search, relevant articles were included based on the authors’ clinical expertise, professional knowledge, and reflective practice.

Inclusion Criteria

The inclusion criteria comprised research focusing specifically on thoracic outlet syndrome (TOS), including its neurogenic (nTOS), arterial (aTOS), and venous (vTOS) subtypes. Studies were included if they evaluated evidence-based diagnostic and therapeutic methods, with a particular emphasis on physiotherapy-based interventions. Eligible studies also investigated structured physiotherapy programs, postural retraining, neural mobilization, stretching, scapular stabilization, and multimodal inpatient rehabilitation. Only articles available on digital platforms such as Google Scholar, ResearchGate, PEDro, MEDLINE, and PubMed were considered, and the search was limited to studies published up to April 2026.

Exclusion Criteria

The exclusion criteria comprised studies that did not provide a strong evidentiary basis for clinical practice. Articles that did not address the diagnostic or therapeutic management of neurovascular bundle entrapment characteristic of TOS were excluded. Studies with significant diagnostic overlap or inconsistent reliability in specialized clinical maneuvers were also excluded because they could hinder the synthesis of accurate evidence. In addition, any articles that failed to meet the predefined eligibility criteria for a comprehensive evaluation of diagnostic frameworks and therapeutic interventions were excluded from the review.

Quality Assessment

Methodological quality and risk of bias were evaluated using study-specific validated tools to address heterogeneity in research designs. Diagnostic reliability was assessed using the QUADAS-2 tool; cross-sectional studies were evaluated using the AXIS tool; and non-randomized interventions were appraised using the ROBINS-I tool [8-10].

Result

**Figure 1:**
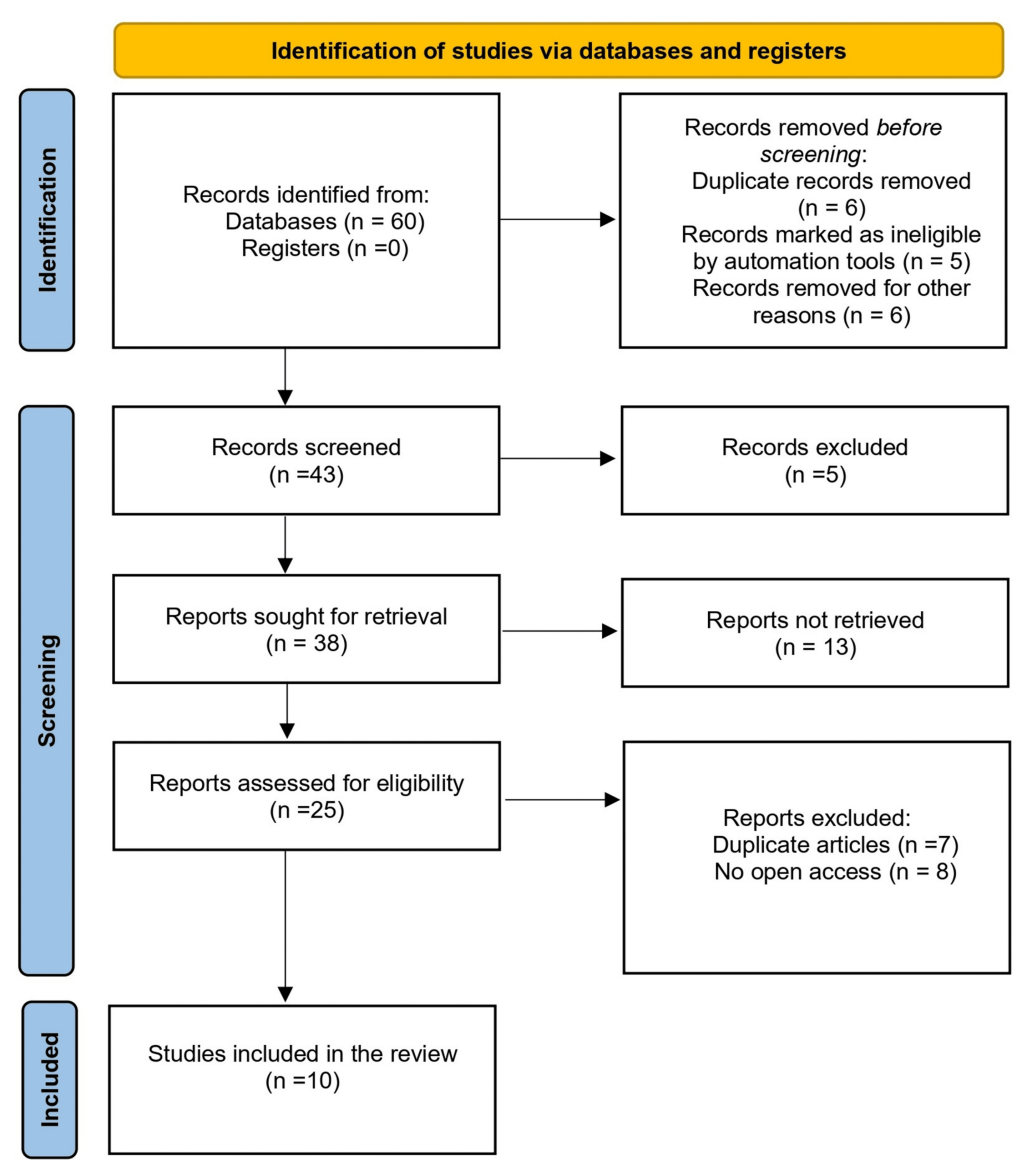
PRISMA flowchart PRISMA: Preferred Reporting Items for Systematic Reviews and Meta-Analyses

A total of 10 articles met the predefined eligibility criteria for this review, providing a comprehensive overview of current diagnostic frameworks and therapeutic interventions for patients with TOS. Figure 1 shows the PRISMA flow chart [7].

The details of the 10 included articles are summarized in Table 1.

**Table 1:** Summary of evidence-based diagnostic and therapeutic methods for TOS TOS: thoracic outlet syndrome, nTOS: neurogenic thoracic outlet syndrome, vTOS: venous thoracic outlet syndrome, aTOS: arterial thoracic outlet syndrome

Study title	Year	Author	Study design	Sample size	Results	Remark
An evaluation of the pain characteristics of patients with thoracic outlet syndrome [11]	2026	Tüzen Tek et al.	Cross-sectional study	66	In 66 TOS patients, longer symptom duration correlated with higher central sensitization and kinesiophobia. Centrally sensitized patients showed greater catastrophizing, kinesiophobia, and alexithymia. Pressure pain thresholds differed in forearm extensors but not in shoulder muscles. Pain severity was similar in surgical and non-surgical patients.	Physiotherapy should emphasize pain neuroscience education, graded exercise, and psychosocial management alongside targeted physical rehabilitation.
Reliability of a standardized ultrasound protocol for the diagnosis of thoracic outlet syndrome [12]	2025	Stegemann et al.	Prospective diagnostic reliability study	51	The standardized ultrasound protocol demonstrated good-to-excellent reliability for diagnosing TOS. Showed high inter-rater and intra-rater reliability, indicating consistency between different examiners. Ultrasound was effective in identifying vascular compression and dynamic changes during positional maneuvers. Improved diagnostic accuracy compared to non-standardized assessment methods.	A standardized ultrasound protocol is a reliable and reproducible tool for diagnosing TOS. It can serve as a non-invasive and cost-effective diagnostic method in clinical practice. Highlights the importance of standardization in imaging techniques to improve diagnostic confidence.
Exploring physiotherapy management of neurogenic thoracic outlet syndrome (nTOS): an online survey of United Kingdom (UK) therapy and medical professionals [13]	2025	O’Sullivan et al.	A cross-sectional online survey	46	The study found wide variation in the assessment and treatment of nTOS among UK clinicians, with common approaches including postural correction, scapular stabilization, stretching, and neural mobilization, highlighting the absence of standardized physiotherapy protocols.	The study highlights a lack of standardized physiotherapy protocols for nTOS and emphasizes the need for evidence-based guidelines. However, findings are limited by self-reported data, potential selection bias, and restricted generalizability beyond the UK clinical population.
Effects of a 3-week inpatient rehabilitation for nTOS on strength, pain, and function: a prospective study [14]	2025	Fouasson-Chailloux et al.	Prospective observational study	220	Among 220 patients, 182 completed evaluations (78% female; mean age 40.7 years). Over 24 weeks, symptomatic limbs showed significant gains in strength, endurance, grip, and pinch. Disability, pain, and sick leave rates markedly decreased, with all improvements statistically significant (p ≤ 0.001).	A three-week inpatient rehabilitation program significantly improved strength, function, pain, and work outcomes in nTOS patients.
Functionality and quality of life analysis of conservative treatment in nTOS [15]	2024	Veloso et al.	Retrospective observational study	56	Conservative physiotherapy markedly improved pain, upper limb function, and quality of life in nTOS patients. Participants experienced enhanced daily activities and functional performance, demonstrating that structured, non-surgical interventions can effectively address symptom burden and improve overall patient outcomes.	The study highlights the effectiveness of conservative physiotherapy for nTOS. While promising, its non-randomized, single-center design and reliance on subjective measures limit the ability to draw definitive conclusions. Controlled trials are needed to confirm efficacy and optimize standardized rehabilitation protocols.
Early results of interventions in patients with vTOS [16]	2024	Elsabbagh et al.	Retrospective multicenter study	23	Twenty-three patients were enrolled, including 16 males (69.5%), with a mean age of 35 ± 9.33 years (range: 23-54 years). Seventeen patients (73.9%) presented with involvement of the right upper limb. All participants were physically active and engaged in daily activities requiring repetitive overhead arm movements. The predominant presenting symptoms included pain or heaviness associated with arm swelling, with or without bluish discoloration of the hands or dilated shoulder veins. Symptoms were exacerbated during shoulder hyperabduction.	This multicenter experience demonstrates that contemporary interventional strategies for vTOS are associated with high technical success, favorable clinical outcomes, and a low complication rate, supporting their safety and effectiveness in the short- to mid-term management of vTOS.
Functional outcome of aTOS treatment [17]	2023	de Kleijn et al.	Retrospective cohort study	20	Treatment of aTOS, including surgical and endovascular interventions, led to substantial improvements in limb function, symptom relief, and overall patient quality of life. The majority of patients reported functional recovery, highlighting the effectiveness of structured, targeted management for arterial compression.	The findings support active intervention for aTOS, but the non-randomized, single-center design and possible confounding factors limit definitive conclusions. Larger, controlled studies are needed to validate outcomes and guide standardized treatment protocols.
Hand strength deficit in patients with nTOS [18]	2021	Fouasson-Chailloux et al.	Prospective cross-sectional study	85	Patients with nTOS showed significant hand strength deficits, particularly in grip and pinch measurements, compared to normative values. The deficits were more pronounced in the affected limb, indicating functional impairment associated with neurogenic compression of the brachial plexus.	The findings highlight the need for targeted rehabilitation focused on hand and upper-limb strengthening in nTOS. Objective hand strength assessment can guide physiotherapy interventions and monitor recovery, though a single-center design and potential confounders limit generalizability.
Feasibility and outcomes of a multidisciplinary care pathway for nTOS: a prospective observational cohort study [19]	2021	Pesser et al.	Prospective observational cohort study	856	Implementation of a multidisciplinary care pathway for nTOS led to significant improvements in pain, upper limb function, and patient-reported quality of life. The pathway was feasible, well-tolerated, and associated with high adherence, indicating potential benefits of coordinated, structured rehabilitation.	The study highlights the value of multidisciplinary rehabilitation in nTOS. However, without a control group and with a single-center cohort, results may be influenced by selection bias and confounding, limiting the strength of causal conclusions. Controlled trials are needed for definitive evidence.
Efficacy of intensive, hospital-based rehabilitation in cases of TOS that failed to respond to private-practice physiotherapy [20]	2020	Thevenon et al.	Retrospective, single-center study	63	The study found that intensive hospital-based rehabilitation significantly improved pain, functional capacity, and quality of life in patients with TOS who did not respond to prior physiotherapy, demonstrating the potential benefit of structured, multidisciplinary inpatient rehabilitation programs.	The findings support intensive rehabilitation for resistant TOS cases; however, the absence of a control group, a retrospective design, and potential selection bias limit causal interpretation and the generalizability of the results.

Assessment of Risk of Bias

To ensure the credibility of the synthesized data, a risk-of-bias evaluation was performed for each selected study. The reliability of the reported outcomes was confirmed by rigorously evaluating all selected literature using validated research tools.

The methodological quality of the included studies varied across diagnostic and observational designs. The diagnostic reliability study by Stegemann et al. was evaluated using the QUADAS-2 tool. It demonstrated a low risk of bias across all four domains: patient selection, index test, reference standard, and flow and timing [12]. Additionally, there were low concerns about applicability, suggesting that the standardized ultrasound protocol is highly reproducible in clinical settings. Table 2 presents the results of the complete quality assessment using QUADAS-2. Appraisal using the 20-item AXIS checklist revealed that while the studies by Tüzen Tek et al. and Fouasson-Chailloux et al. were methodologically rigorous in their reporting of results and ethical standards, both lacked explicit sample size justification and were susceptible to selection bias due to their clinical recruitment methods [11,18]. O’Sullivan et al. demonstrated the highest vulnerability to bias, specifically regarding sampling frame and non-response bias, as the study utilized a non-representative snowball sampling method for its online survey [13]. Table 3 presents the results of the complete quality assessment using the AXIS tool. For non-randomized intervention studies, the risk of bias varied significantly. Studies by Fouasson-Chailloux et al. and Pesser et al. were rated as having a moderate overall risk of bias [14,19]. In contrast, several retrospective studies were rated as having a serious risk of bias, primarily due to confounding, participant selection issues, and the absence of control groups. Table 4 presents the results of the complete quality assessment using the ROBINS-I tool.

**Table 2:** Quality assessment using four domains of the QUADAS-2 tool ROB: risk of bias

Study	Patient selection (ROB)	Index test (ROB)	Reference standard (ROB)	Flow and timing (ROB)	Patient selection (applicability)	Index test (applicability)	Reference standard (applicability)	Overall ROB
Stegemann et al. [12]	Low	Low	Low	Low	Low concern	Low concern	Low concern	Low

**Table 3:** Quality assessment of included cross-sectional studies using the AXIS tool

AXIS appraisal question	Tüzen Tek et al. [11]	O’Sullivan et al. [13]	Fouasson-Chailloux et al. [18]
Were the aims/objectives of the study clear?	Yes	Yes	Yes
Was the study design appropriate for the aims?	Yes	Yes	Yes
Was the sample size justified?	No	No	No
Was the target population clearly defined?	Yes	Yes	Yes
Was the sampling frame appropriate?	Yes	No	Yes
Was the selection process representative?	No	No	No
Were measures taken to address non-responders?	Yes	No	Yes
Were the outcome variables measured appropriately?	Yes	Yes	Yes
Were the tools piloted/published previously?	Yes	Yes	Yes
Was statistical significance clearly determined?	Yes	Yes	Yes
Were the methods described in enough detail?	Yes	Yes	Yes
Were the basic data adequately described?	Yes	Yes	Yes
Is there concern regarding non-response bias?	No	Yes	No
Was the info about non-respondents described?	No	No	No
Were the results internally consistent?	Yes	Yes	Yes
Were all method-defined results presented?	Yes	Yes	Yes
Were discussions and conclusions justified?	Yes	Yes	Yes
Were the study limitations discussed?	Yes	Yes	Yes
Were funding/conflicts of interest reported?	Yes	Yes	Yes
Was ethical approval/informed consent attained?	Yes	Yes	Yes

**Table 4:** Risk of bias assessment of non-randomized interventions using the ROBINS-I tool

Study	Bias due to confounding	Bias in selection of participants	Bias in classification of interventions	Bias due to deviations from intended interventions	Bias due to missing data	Bias in measurement of outcomes	Bias in selection of the reporter results	Overall risk of bias
Fouasson-Chailloux et al. [14]	Moderate risk	Moderate risk	Low risk	Low risk	Low risk	Moderate risk	Moderate risk	Moderate risk
Veloso et al. [15]	Moderate risk	Moderate risk	Low risk	Moderate risk	Moderate risk	Moderate risk	Low risk	High risk
Elsabbagh et al. [16]	Serious risk	Moderate risk	Low risk	Moderate risk	Moderate risk	Moderate risk	Moderate risk	Serious risk
De Kleijn et al. [17]	Moderate risk	Moderate risk	Low risk	Low risk	Moderate risk	Moderate risk	Moderate risk	Serious risk
Pesser et al. [19]	Moderate risk	Moderate risk	Low risk	Low risk	Moderate risk	Moderate risk	Moderate risk	Moderate risk
Thevenon et al. [20]	Serious risk	Moderate risk	Low risk	Moderate risk	Moderate risk	Moderate risk	Moderate risk	Serious risk

Discussion

TOS, characterized by a wide range of causes, occurs when the neurovascular bundle is mechanically entrapped within the thoracic outlet. This review highlights evidence-based diagnostic and therapeutic approaches, emphasizing physiotherapy as a first-line conservative management strategy. Across the 10 included studies, a range of interventions were investigated, including structured physiotherapy programs, stretching, neural mobilization, postural retraining, scapular stabilization, and multimodal inpatient rehabilitation. The synthesized evidence demonstrates that such interventions are effective in alleviating symptoms and restoring physical performance, specifically targeting deficits in strength and daily functioning in the nTOS population. For example, O’Sullivan et al. highlighted variability in UK physiotherapy practice, noting that postural correction, scapular stabilization, and neural mobilization are commonly used but lack standardized protocols [13]. Similarly, Fouasson-Chailloux et al. reported marked improvements in shoulder and hand strength, endurance, pain, and disability scores following a three-week inpatient rehabilitation program, with effects persisting up to 24 weeks [14].

Dengler et al. identify nTOS as a condition defined by the constriction of the brachial plexus within the thoracic outlet. Their findings highlight that this pressure leads to significant upper-limb symptoms, such as numbness and reduced strength, particularly during arm movement. The authors discuss epidemiology, varied clinical presentations, diagnostic challenges, and the importance of clinical assessment supported by imaging and electrodiagnostics. Management includes conservative therapy (physical therapy and postural correction) and, in selected cases, surgical decompression to relieve neural compression and improve function [5].

According to Stegemann et al., the reliability of a structured ultrasound protocol for diagnosing aTOS was evaluated in 51 patients with high clinical suspicion [12]. Two sonographers (one experienced, one less experienced) independently performed the standardized ultrasound, with the experienced examiner as reference. The protocol was feasible in all patients, and inter-rater agreement was very good (κ = 0.820), indicating that the standardized approach yields consistent results between examiners. The study supports the use of standardized ultrasound as a reliable diagnostic tool for aTOS [12,13].

The systematic review by Garraud et al. examines TOS in athletes, highlighting its prevalence, risk factors, diagnosis, and management [21]. TOS is increasingly recognized among overhead athletes, such as swimmers, baseball pitchers, volleyball players, and weightlifters, in whom repetitive arm elevation and strenuous activity contribute to neurovascular compression. Although nTOS is most prevalent in the sporting world, the repetitive physical demands of high-level athletics also lead to a notable incidence of venous and arterial complications within the neurovascular bundle [18-20].

Risk factors include repetitive overhead movements, muscular hypertrophy, poor posture, and anatomical variations such as cervical ribs. Symptoms often present as upper limb pain, numbness, weakness, fatigue, and decreased athletic performance. In vTOS, athletes may exhibit swelling, cyanosis, or thrombosis. The literature highlights the difficulty of confirming a TOS diagnosis, noting that its presentation is often nonspecific. This creates a diagnostic overlap with various upper extremity disorders, requiring careful differentiation from shoulder-related pathologies and nerve root compression [20,21]. The diagnostic process for TOS is best navigated through a combination of physical clinical evaluation, advanced imaging techniques, and various functional provocative maneuvers.

Conservative management, particularly physiotherapy focusing on postural correction, stretching, strengthening, and activity modification, is the first-line treatment. Surgical intervention may be required in refractory or vascular cases. The review concludes that early identification and multidisciplinary management are crucial for preventing complications and enabling a safe return to sport [21].

Camporese et al. synthesize current evidence on TOS and present a retrospective analysis of 324 patients from two Italian hospitals who were followed for five years. The study classifies TOS into nTOS (90-95%), vTOS (3-5%), and aTOS (1-2%) subtypes, noting that diagnosis remains challenging due to the nonspecific nature of symptoms. The authors advocate for a multimodal diagnostic strategy, specifically highlighting the utility of dynamic color-coded Doppler ultrasonography (CCDU) for vascular assessment. Their report emphasizes that specialized physical therapy and rehabilitation programs effectively relieve symptoms for most patients, while surgical decompression is reserved for severe or refractory cases [22].

Hooper et al. emphasize that conservative, non-surgical management is the primary treatment for TOS. Rehabilitation focuses on correcting identified mechanical dysfunctions through first-rib mobilization, restoration of the costoclavicular space, and diaphragmatic breathing to minimize scalene overactivity. The study highlights the importance of patient education on posture and the "release phenomenon," in which symptoms may temporarily intensify as nerve function normalizes. Surgery, typically first-rib resection or scalenectomy, is reserved for vascular cases or neurogenic patients who fail intensive conservative trials or exhibit progressive neurological deficits [23].

Elshinnawy et al. investigated the impact of osteopathic manipulative techniques (OMT) on pain and respiratory function in 40 adults with TOS. Participants were randomized to a traditional physical therapy group or a combined group that received OMT once weekly for three months. Results demonstrated that the OMT group achieved significant improvements in respiratory parameters, including maximal inspiratory and expiratory pressures, forced vital capacity, and forced expiratory volume. Additionally, the OMT group reported a marked reduction in neck and upper arm pain, suggesting that manual osteopathic techniques effectively complement standard physical therapy for TOS rehabilitation [24].

Dengler et al. define nTOS as the mechanical compression of the brachial plexus within the thoracic outlet. Clinical presentation typically includes significant upper-limb numbness and weakness, which often worsen with arm movement. The authors emphasize that diagnosis is complex and requires a thorough clinical assessment supported by imaging and electrodiagnostics. First-line management focuses on conservative strategies, specifically physical therapy and postural correction. In selected refractory cases, surgical decompression is utilized to relieve neural pressure, restore function, and improve the patient's quality of life [25].

Clinical Implications

Effective management of TOS hinges on early recognition, particularly when upper-limb discomfort fluctuates with activity or position rather than following a clear nerve-root distribution. To guide recovery, clinicians should use objective assessments, such as handgrip and pinch strength, to monitor progress and guide treatment through structured physiotherapy programs that emphasize postural correction, scapular stabilization, and neural mobilization. Given that recovery is often complicated by psychosocial factors like central sensitization and kinesiophobia, a biopsychosocial approach is essential, integrating pain education and graded exposure into the treatment plan. This is best achieved through a multidisciplinary framework that combines physiotherapy, occupational therapy, and manual techniques while prioritizing patient education on activity modification and self-management to ensure long-term symptom control and prevent recurrence.

Limitations

The limitations of this systematic review stem from a significant lack of standardized protocols for both diagnostic evaluation and physiotherapy interventions, leading to inconsistent clinical practices across the included literature. Methodologically, the review is constrained by a relatively small sample of 10 articles, several of which exhibit a "serious" overall risk of bias due to confounding and participant selection issues. Furthermore, many of the studies analyzed use retrospective, single-center designs and lack control groups, which limits the generalizability of the findings and the ability to establish definitive causal links between treatments and outcomes. Finally, the inherent diagnostic complexity of TOS, characterized by overlapping symptoms with other pathologies and the inconsistent reliability of specialized maneuvers, remains a major hurdle in ensuring the accuracy and specificity of the synthesized evidence.

Future Scope

Future research should prioritize large-scale, multicenter randomized controlled trials to establish definitive causal relationships and mitigate the biases inherent in current retrospective and single-center studies. A critical focus must be the development and validation of standardized, evidence-based clinical protocols to address the significant variability currently observed in diagnostic evaluations and physiotherapy interventions. Additionally, to overcome the diagnostic complexity and inconsistent reliability of specialized clinical maneuvers, future studies should advance the validation of objective diagnostic tools, such as standardized ultrasound, alongside functional outcome metrics like handgrip and pinch strength. Finally, given that long-term success is maximized by addressing psychological barriers such as kinesiophobia and central sensitization, further investigation is warranted into the long-term clinical efficacy of multidisciplinary, biopsychosocial care pathways that formally integrate pain neuroscience education with physical rehabilitation.

## Conclusions

This review concludes that structured physiotherapy encompassing postural correction, scapular stabilization, and neural mobilization is highly effective as a primary conservative intervention for improving pain and upper limb function. Furthermore, long-term success is maximized by adopting a multidisciplinary, biopsychosocial approach that integrates pain neuroscience education to address psychological barriers like kinesiophobia and central sensitization. While surgical decompression remains an option for refractory or severe vascular cases, the prevailing evidence emphasizes that early identification, comprehensive patient education, and the development of standardized rehabilitation protocols are essential for optimizing patient outcomes and preventing symptom recurrence.
